# Momentum‐Space Imaging of Ultra‐Thin Electron Liquids in δ‐Doped Silicon

**DOI:** 10.1002/advs.202302101

**Published:** 2023-07-19

**Authors:** Procopios Constantinou, Taylor J. Z. Stock, Eleanor Crane, Alexander Kölker, Marcel van Loon, Juerong Li, Sarah Fearn, Henric Bornemann, Nicolò D'Anna, Andrew J. Fisher, Vladimir N. Strocov, Gabriel Aeppli, Neil J. Curson, Steven R. Schofield

**Affiliations:** ^1^ London Centre for Nanotechnology University College London London WC1H 0AH UK; ^2^ Department of Physics and Astronomy University College London London WC1E 6BT UK; ^3^ Photon Science Division Paul Scherrer Institut Villigen‐PSI 5232 Switzerland; ^4^ Department of Electronic and Electrical Engineering University College London London WC1E 7JE UK; ^5^ Advanced Technology Institute University of Surrey Guildford GU2 7XH UK; ^6^ Department of Materials Imperial College of London London SW7 2AZ UK; ^7^ Institute of Physics Ecole Polytechnique Fédérale de Lausanne (EPFL) Lausanne 1015 Switzerland; ^8^ Department of Physics ETH Zürich Zurich 8093 Switzerland; ^9^ Quantum Center Eidgenössische Technische Hochschule Zurich (ETHZ) Zurich 8093 Switzerland

**Keywords:** 2DEG, ARPES, arsenic in silicon, delta layer, silicon, soft X‐ray angle‐resolved photoelectron spectroscopy (soft X‐ray ARPES)

## Abstract

Two‐dimensional dopant layers (δ‐layers) in semiconductors provide the high‐mobility electron liquids (2DELs) needed for nanoscale quantum‐electronic devices. Key parameters such as carrier densities, effective masses, and confinement thicknesses for 2DELs have traditionally been extracted from quantum magnetotransport. In principle, the parameters are immediately readable from the one‐electron spectral function that can be measured by angle‐resolved photoemission spectroscopy (ARPES). Here, buried 2DEL δ‐layers in silicon are measured with soft X‐ray (SX) ARPES to obtain detailed information about their filled conduction bands and extract device‐relevant properties. This study takes advantage of the larger probing depth and photon energy range of SX‐ARPES relative to vacuum ultraviolet (VUV) ARPES to accurately measure the δ‐layer electronic confinement. The measurements are made on ambient‐exposed samples and yield extremely thin (< 1 nm) and dense (≈10^14^ cm^−2^) 2DELs. Critically, this method is used to show that δ‐layers of arsenic exhibit better electronic confinement than δ‐layers of phosphorus fabricated under identical conditions.

## Graphical abstract

Soft X‐ray angle‐resolved photoemission spectroscopy (SX‐ARPES) measurements of metallic dopant layers in silicon reveal that arsenic δ‐layers surpass phosphorus ones in two‐dimensionality. The thinnest arsenic layer boasts an electronic width of 0.45 nm and the ability to engineer such ultra‐thin, high carrier density layers represents yet another capability that can be exploited for new nano‐ and quantum‐electronic applications in silicon.

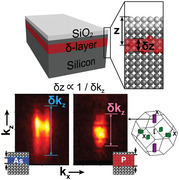

## 1 Introduction

**Figure 1:**
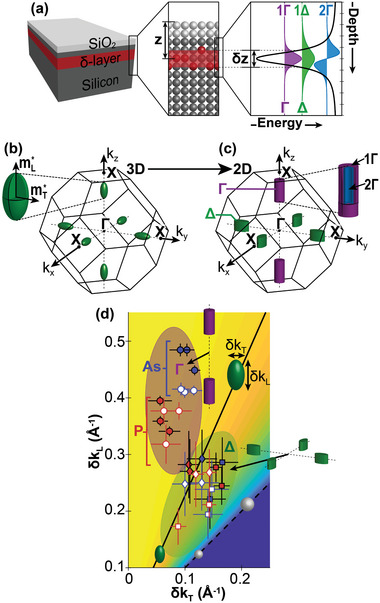
Sample schematic and the evolution of the silicon conduction valleys vs δ‐layer confinement. a) Schematic representation of our δ‐layer samples, with a native oxide that forms due to ambient exposure. The silicon overgrowth thickness, *z*, is indicated, as is the electronic thickness of the δ‐layer, δ*z*. The δ‐layer creates an approximately V‐shaped potential well in the plane perpendicular to the δ‐layer, which quantizes the out‐of‐plane and in‐plane conduction valleys into a series of subbands denoted as *n*Γ and *n*Δ, respectively. The tic‐marks on the depth axis indicate 1 nm steps. b) Evolution of the silicon conduction valleys from 3D (six degenerate, ellipsoidal valleys) to c) 2D (4 Δ‐valleys + 2 Γ‐valleys). d) Plot of the transverse (δ*k*_T_) versus longitudinal (δ*k*_L_) extent of the *k_x_* (diamonds, green region), *k*_y_ (squares, green region) and *k*_z_ valleys (circular markers, purple region). The filled and hollow markers represent data from 2 and 3 nm deep δ‐layers respectively. The solid black line indicates the expected valley morphology for bulk, 3D silicon, whose gradient is equal to the mass anisotropy of silicon. The colored background represents the eccentricity of the ellipsoid, which spans from zero (blue) to one (yellow).

Two‐dimensional (2D) quantum‐confined electronic systems have long been venues for discoveries in fundamental physics and the development of new devices.^[^ ^1^ ^]^ Technological 2D systems have traditionally consisted of planar heterostructures and field‐effect devices, particularly in compound semiconductors.^[^ ^2^ ^]^ In recent years, there has similarly emerged strong interest in 2D electron states in van der Waals systems, such as graphene, and the transition metal dichalcogenides for future nanoscale and quantum‐electronic devices.^[^ ^3^, ^4^, ^5^ ^]^ Understandably, there is also strong interest in fabricating 2D electron states in the world's leading technological semiconductor, silicon. This is largely driven by the requirements of proposed nano‐ and quantum‐electronic applications employing atomically abrupt dopant profiles, e.g., the famed Kane solid‐state quantum computer and related designs.^[^ ^6^, ^7^, ^8^ ^]^ 2D electron states can be created in silicon via so‐called δ‐doping, which involves the physical^[^ ^9^ ^]^ or chemical^[^ ^10^ ^]^ deposition of dopant atoms onto a silicon surface, followed by silicon overgrowth to produce sharp, 2D doped layers (**Figure** **1a**). At high doping concentrations, such δ‐layers yield quantum‐confined 2D conductive planes with electronic properties significantly different to those of the bulk silicon host.^[^ ^11^ ^]^

The thinnest δ‐layers prepared in silicon to date have relied on the chemical delivery of phosphorous,^[^ ^10^ ^]^ arsenic,^[^ ^12^ ^]^ or boron,^[^ ^13^ ^]^ with the resulting out‐of‐plane atomic distributions of dopant atoms having ≈1 nm thicknesses.^[^ ^14^, ^15^, ^16^, ^17^ ^]^ The electronic thicknesses of these layers have also been estimated using quantum magnetoresistance,^[^ ^18^ ^]^ with similar results.^[^ ^19^ ^]^ Such thicknesses are comparable to the wavelength of the conduction electrons, and the corresponding energy level quantization was observed in planar junction tunneling spectroscopy more than three decades ago.^[^ ^9^, ^20^, ^21^ ^]^ Vacuum ultraviolet angle‐resolved photoemission spectroscopy (VUV‐ARPES) measurements of phosphorous δ‐layers in silicon have also revealed quantized states, yet the origin of these quantized states was incorrectly attributed to the more exotic degeneracy lifting mechanism, valley interference.^[^ ^22^, ^23^, ^24^, ^25^ ^]^ To justify the anomalously large valley splitting energies reported, the authors cited density functional theory (DFT) calculations that were made for perfectly ideal, one‐atom‐thick δ‐layers. However, DFT calculations of δ‐layers with even a single atom deviation from a perfectly thin δ‐layer show the valley splitting reduces to ≈1 meV.^[^ ^26^ ^]^ Such small valley‐splitting energies cannot presently be observed in ARPES measurements, and it has since been acknowledged that the observed splitting is due to confinement,^[^ ^27^, ^28^ ^]^ as first suggested in the 1980s.^[^ ^9^, ^20^, ^21^ ^]^ Moreover, as discussed in Refs. ^[^ ^22^, ^23^ ^]^ the short inelastic mean free path of the ejected electrons in VUV‐ARPES (λ_*e*_ ≈ 0.5 nm) means the signal for previous ARPES measurements^[^ ^23^, ^28^, ^29^ ^]^ does not directly originate from the δ‐layer (that is up to 4λ_e_ beneath the surface), but is instead a near‐surface resonance enhancement that enables only a small fraction of the wavefunction to be probed.^[^ ^23^ ^]^ Furthermore, because VUV‐ARPES has limited momentum resolution along the surface normal, it was impossible to measure a corresponding momentum spread whose inverse would be the key parameter of the 2DEL, namely the electronic thickness, from which the origin and level quantization of the 2DEL can be deduced.

In this paper, we report comprehensive soft X‐ray ARPES (SX‐ARPES) measurements of δ‐layers in silicon. The high photon energies of SX‐ARPES (*h*ν = 300–1600 eV) give access to a much longer electron mean free path (λ_e_ ≈ 2 nm), which permits the extraction of electrons from depths of several nanometers beneath the surface.^[^ ^30^ ^]^ This enables us to directly probe δ‐layers underneath the native surface oxide of samples exposed to ambient after their fabrication, while maintaining a very sharp out‐of‐plane *k*_z_ momentum resolution, Δ*k*_z_, which is equal to $\Delta k_{z}=\lambda_{e}^{-1}$.^[^ ^31^ ^]^ Our experiments therefore differ qualitatively from the previous VUV‐ARPES.^[^ ^22^, ^23^, ^24^, ^25^ ^]^ We present, for the first time, energy and momentum maps resolved with high momentum resolution in the plane perpendicular to the δ‐layer, revealing the detailed δ‐layer band structure in the *k*_z_‐*k*_∥_ plane. Our measurements conclusively demonstrate that the δ‐layer band structure is non‐dispersive in the plane perpendicular to the δ‐layer in a manner significantly more convincing than a previous attempt using VUV‐ARPES *k*_z_‐binding energy scans.^[^ ^22^ ^]^ Moreover, exactly as for photoemission tomography of molecules,^[^ ^32^, ^33^, ^34^ ^]^ our *k*_z_ momentum dependencies are related via a Fourier transform to electron densities in real space, and thus measure directly the real‐space thicknesses of the occupied quantized electronic states that constitute the 2DEL. We apply this method to investigate the optimization of δ‐layer electronic thickness in silicon, and to compare δ‐layers fabricated with arsenic and phosphorus. We show that the electrons in arsenic δ‐layers are significantly more confined than in phosphorus δ‐layers prepared under identical conditions, and we determine the carrier density via a Luttinger analysis of the Fermi surface.

Our SX‐ARPES experiments feature an X‐ray spot size of (10 × 73) µm^2^, which is comparable to the size of the Hall‐bars used for quantum magnetotransport measurements. Next‐generation light sources together with new optics will enable SX‐nanoARPES with better energy resolution and sub‐micron spot sizes,^[^ ^35^ ^]^ thus providing a tool complementary to X‐ray inspection of integrated circuit morphology^[^ ^36^, ^37^ ^]^ and chemical composition in the sense that it will image the electrons actually switched in devices. While such ARPES measurements have already been conducted in the UV regime,^[^ ^38^, ^39^ ^]^ extension to the SX regime will offer an enhanced bulk sensitivity for probing buried heterostructures or interfaces. Although scanning microwave microscopy^[^ ^40^ ^]^ also images the conduction electrons in devices, it does not yield their 3D momentum distribution. However, SX‐nanoARPES, along with the methods and analysis we present here, can do so, greatly expanding the possibilities for characterizing semiconductor nanostructures and devices.

## 2 Background

The dynamic behavior of conduction electrons in bulk silicon is determined by a set of six degenerate conduction band valleys, with minima at equivalent points in reciprocal space along the <100> directions.^[^ ^41^ ^]^ Bulk electron doping causes these valleys to become occupied and, at high doping levels, will result in ellipsoidal Fermi surfaces, one around each minimum (Figure 1b). However, when electrons are confined to 2D planes, as for δ‐doping, the Bloch wavevector component in the *k*_z_ direction is no longer a good quantum number, and the energy becomes quantized into discrete levels, *E*_n_. The in‐plane wavevector components *k*_x_ and *k*_y_ remain good quantum numbers and the electronic states can be described using the formalism of effective mass theory.^[^ ^42^ ^]^

According to elementary quantum mechanics, the degree of confinement is governed by the potential created by the δ‐layer, the effective mass of the electrons, and the number of wavefunction nodes. Since the δ‐doping breaks the degeneracy of the six valleys, the two valleys centered at *k*_x_ = *k*_y_ = 0 are characterized by a single, in‐plane, transverse effective mass and the quantized states are correspondingly labeled *n*Γ (where *n* is the subband number), while the remaining four in‐plane valleys are characterized by in‐plane longitudinal and transverse effective masses and are labeled *m*Δ (where *m* is the subband number).^[^ ^43^, ^44^, ^45^ ^]^ Subsequently, in the direction of quantization the *n*Γ and *m*Δ subbands derive from bands with a heavy and light effective mass respectively, leading to different spectra for states derived from different valleys. The right‐hand panel of Figure 1a shows a self‐consistent Schrödinger–Poisson model of how the *n*  =  1 and *n*  =  2 wavefunctions (labeled 1Γ and 2Γ) for electrons with a heavy mass bracket the *m*  =  1 wavefunction (labeled 1Δ) for the lighter, and hence less confined, electron; the simulation in Figure 1a was performed using the electron density and electronic thickness extracted from our SX‐ARPES measurements of a 2 nm overgrown arsenic δ‐layer, as described below. Moreover, our calculations treat the *n*Γ and *m*Δ subbands as standing wave solutions that originate from the superposition of two plane waves moving with ±*k*_z_ momenta, confined by the boundary of the δ‐layer and in the absence of so‐called valley interference.^[^ ^11^ ^]^

In practice, the δ‐layer wave function is characterized by an envelope function in the z‐direction that decays with distance away from the δ‐layer, combined with an oscillatory Bloch wave component established by the bulk conduction states from which the δ‐layer is derived. The Fourier spectrum of such a state is peaked about the values of *k*_z_ corresponding to its Bloch wave origins and is oscillatory in *k*_z_ at multiples of the reciprocal lattice vector.^[^ ^30^, ^46^, ^47^ ^]^ Thus, the Fermi surface picture of Figure 1b is transformed by the replacement of conduction ellipsoids with states that do not disperse in *k*_z_, and can be visualised, from the standpoint of an ARPES experiment, as being cylindrical or elliptic‐cylindrical in shape (Figure 1c); the extent of these states in *k*_z_ is inversely proportional to the electronic (not chemical) real‐space thickness of the δ‐layer.^[^ ^25^, ^30^ ^]^ A 2D system confined along *z* by an infinitely deep and infinitesimally narrow potential would yield states with infinitely long profiles along *k*_z_, while at the other extreme, for a fully 3D doped system, the states should return to reside within the ellipsoidal Fermi‐surfaces shown in Figure 1b. For real layers of some finite thickness, a phenomenological equation for the thickness of the layer is^[^ ^30^ ^]^:

(1) $$\delta z=\frac{1}{\delta k_{z}-\delta k_{\infty }}\;$$

where δ*k*_z_ is the extent of the 2D valley state in *k*_z_, and δ*k*_∞_ is the corresponding length of the state for the same electron doping level in the absence of 2D confinement. We determine δ*k*_z_ and δ*k*_∞_ experimentally from our SX‐ARPES data by measuring the longitudinal extent of the out‐of‐plane (Γ) valley, and the in‐plane (Δ) valleys respectively. Careful measurement of these quantities and application of Equation (1) thus produces a direct measure of the electronic thickness, δ*z*, of the δ‐layers.

Figure 1d summarizes our results for the electronic thickness of the δ‐layer. Here we show the longitudinal extent of the in‐plane and out‐of‐plane valleys versus their transverse extent. The data clusters into two groups, for the Γ and Δ valleys, respectively. In particular, the Δ valleys lie along a straight line characterising the ellipsoidal shape of the bulk silicon conduction band valleys (as set by the ratio of the bulk longitudinal and transverse effective masses). In stark contrast, the Γ valleys appear elongated in the longitudinal direction and are therefore grouped together in the top left of the plot. This lengthening of the states in *k*_z_ is characteristic of 2D electronic states, to be discussed further below.

## 3 Results

### 3.1 δ‐Layer Carrier Density and Fermi‐Surface Measurements

We fabricated δ‐layer samples using either phosphorus or arsenic as the dopant species. The Experimental Section gives details of the sample preparations. Secondary ion mass spectrometry (SIMS) and Hall effect measurements confirmed the anticipated highly peaked dopant distributions and dopant electrical activations for all samples (see Supporting Information).

**Figure 2:**
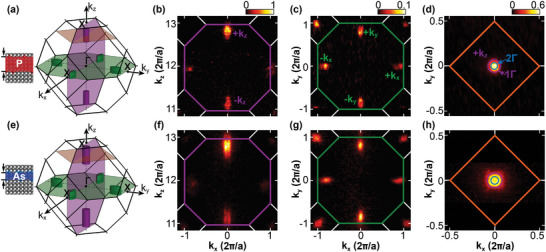
Fermi‐surface measurements of (upper row) phosphorous and (lower row) arsenic δ‐layers with 2 nm silicon overgrowth. a,e) Schematic representations of the measured six conduction valleys of silicon embedded within the bulk fcc Brillouin zone, indicating 2D behavior. Fermi surface slices for the phosphorous and arsenic δ‐layer samples are shown along the following planes: b,f) *k*_x_‐*k*_z_ and c,g) *k*_x_‐*k*_y_ through the zone center (Γ), d,h) *k*_x_‐*k*_y_ through the center of the upper *k*_z_ valley (see also the color‐coded slices on panels a,b). Fermi surfaces are integrated from −50 meV to *E*_F_. In panel (d,h), the 1Γ and 2Γ states are denoted, based on the fits acquired in Figure 4.

In **Figure** **2**, we show the SX‐ARPES Fermi surface maps acquired from a phosphorus (Figure 2a–d) and an arsenic (Figure 2e–h) δ‐layer. The schematic Brillouin zone diagrams at the left of the figure illustrate the planes through which each of the Fermi surface slices have been taken: Figure 2b,f shows *k*_x_–*k*_z_ slices that cut through two Γ and two Δ valleys, illustrated by the purple plane in the schematics. Figure 2c,g,d,h shows *k*_x_–*k*_y_ slices at different *k*_z_ values, as indicated by the green and orange planes in the schematics, respectively.

The degeneracy breaking due to δ‐layer confinement is readily apparent for both samples: the four Δ‐valleys in the *k*_x_–*k*_y_ slices (Figure 2c,g) are uniform in size and shape, as expected, while in the *k*_x_–*k*_z_ slices (Figure 2b,f) we find the two Γ‐valleys (at ± *k_z_*) appear significantly larger and brighter than the Δ‐valleys. The main difference in intensity occurs because of the different in‐plane effective masses of the two types of valleys, resulting in a different electronic density of states and hence measured spectral weights.^[^ ^44^ ^]^

We can determine the 2D carrier density of the samples by analyzing the area enclosed by each valley in the *k*_x_–*k*_y_ plane; in other words, determining the total area enclosed by the four Δ valleys in Figure 2c,g and also the *k*_x_–*k*_y_ slice through the two Γ valleys, one of which is shown in Figure 2d,h. We find that the resulting total carrier density for all samples lie within the range (0.88 ± 0.10) × 10^14^ cm^−2^, consistent with Hall effect measurements for all but one of the samples considered (see Supporting Information). This concurs with our expectations, as at the self‐saturation limit of δ‐doping, one in every four silicon (001) surface atoms is replaced with a dopant, corresponding to a density of ≈ 1.4 × 10^14^ cm^−2^.^[^ ^48^ ^]^ We attribute the reduced measured carrier density to the deactivation of some donors via effects such as clustering (particularly for arsenic)^[^ ^49^, ^50^ ^]^ and chemical interaction with oxygen atoms where the native oxidation of the surface and δ‐layer overlap. Furthermore, we find that the carriers are equally distributed within the Γ and Δ subbands (see Supporting Information), in agreement with the theoretical predictions of Ref. [42] and our own Schrödinger–Poisson modeling (Figure 1a), in contrast to previous VUV‐ARPES that showed an unoccupied Δ band.^[^ ^27^ ^]^

### 3.2 δ‐Layer Thickness Determination

**Figure 3:**
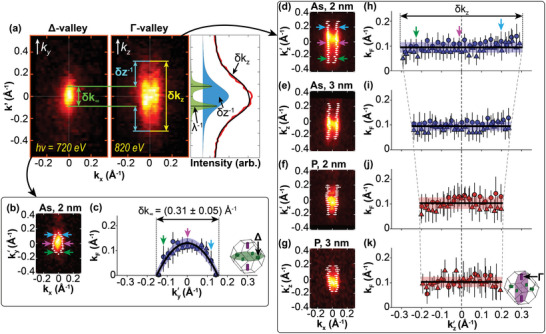
Extracting δ‐layer confinement from the longitudinal span of the **Δ**‐ and **Γ**‐valleys. a) Visual representation of Equation (1), which is applied to extract the δ‐layer confinement, δ*z*, from the longitudinal extent of the Δ‐ and Γ‐valleys. δ*k*_∞_ represents the longitudinal FWHM of the Δ‐valley along the *k*_y_‐axis, which is broadened by the intrinsic mean free path (MFP), λ; δ*k*_z_ is the longitudinal FWHM of the Γ‐valley along the *k*_z_‐axis, which includes both the MFP and confinement broadening. The line profile shows the Lorentzian deconvolution process (see Supporting Information for more details), allowing the δ‐layer confinement to be extracted from the *k*_z_ response of the Γ‐valley; a reasonable fit (black) to the data (red) can be achieved by convolving the green and blue contributions. b) *k*_x_‐*k*_y_ Fermi surface and c) *k*_F_ vs *k*_y_ for the 2 nm arsenic δ‐layer sample, where $k_{y}^{\prime }=k_{y}-\;$0.94 Å^–1^. d–g) *k*_x_‐*k*_z_ Fermi surfaces (*h*ν = 350 – 410 eV, integrated from −50 meV to *E*_F_) for 2 and 3 nm phosphorous and arsenic δ‐layers, as indicated. White dots indicate the cusps of the parabola in *k*_x_ at each value of *k*_z_, where $k_{z}^{\prime }=k_{z}-$10.18 Å^–1^. h–k) Plots of *k*_F_ as a function of *k*_z_ extracted from panels (a–d), whereas the triangle data points are taken from the same valley, but at a higher photon energy (≈ 820 eV). The inset of (c) and (k) indicates the valley that is probed; the green, pink, and blue arrows in panels (b,c) and (d,h) offer a guide to the eye. The best fit line is shown in black, with the shaded areas indicating the 1σ fit confidence.

As discussed above, an electronically 2D δ‐layer should be dispersionless in *k*_z_, and therefore its Γ valley should be a regular cylinder, rather than ellipsoidal. In addition, the extent of the state in *k*_z_ provides a direct measure of the confinement thickness of the state. With this in mind, we have performed a quantitative analysis of four δ‐layer samples, as shown in **Figure** **3**. Two of the samples were phosphorous δ‐layers and two were arsenic δ‐layers, and for each dopant species we have performed a nominal silicon overgrowth of 2 and 3 nm. Figure 3a summarizes our approach to determine the δ‐layer confinement from the high‐resolution Fermi surface maps of the +*k*_z_ Γ‐valleys (Figure 3d–g), and a comparable +*k*_y_ Δ‐valley (Figure 3b). We note that measurements were also made on samples overgrown with 1 and 4 nm of silicon. For the former, no conduction states were observed, which we attribute to the complete oxidation of the δ‐layer when the sample was exposed to ambient for transport to the synchrotron. For the latter, the spectral intensity of the conduction states became very weak, due to the electron escape depth being smaller than the δ‐layer depth, making the analysis extremely difficult.

We have used an automated procedure to extract the edges of the +*k*_z_ valleys: for each horizontal line‐profile cut of the Fermi surface, we find the edges of the valleys, whose positions are shown as pairs of white dots on Figure 3d–g. For the arsenic δ‐layer samples, two distinct peaks in each line‐cut along *k*_x_ are resolved and tracked. These two peaks correspond to the cusps of the parabolic dispersion of the electrons in *k*_x_. For the phosphorous δ‐layer samples, the peaks along *k*_x_ could not be resolved directly, so instead the FWHM was measured. For each value in *k*_z_, the separation between these two dots along the *k*_x_ direction gives a measure of the Fermi wavevector, *k*_F_, and these values of *k*_F_ are plotted against *k*_z_ in the corresponding panels Figure 3h–k. For each of the four δ‐layer samples, we see that *k*_F_ remains constant as a function of *k*_z_ to within the uncertainties of our measurements, demonstrating that each of the four samples are dispersionless in *k*_z_, as expected. For comparison, in Figure 3b,c, we apply the same analysis to one of the in‐plane Δ valleys to plot *k*_F_ as a function of *k*_y_. Here, we see that *k*_F_ is not constant, but instead exhibits the expected dispersion corresponding to the longitudinal effective mass, from which we extract a value of (0.90  ±  0.05)*m*_e_, in agreement with its accepted value.^[^ ^51^ ^]^

The analysis in Figure 3h–k provides a measure of the length of these features in *k*_z_, i.e., δ*k*_z_. We obtain the corresponding 3D width, δ*k*_∞_ from the analysis of the in‐plane valley in Figure 3c. Using these values, we then extract the real space electronic thickness of the δ‐layer using Equation (1). We find that for the arsenic δ‐layer samples, δ*z* = 5.4 ± 0.1 Å, whereas for the phosphorus δ‐layer samples, δ*z* = 9.7 ± 4.1 Å. A summary of the δ‐layer thickness measurements using SIMS and SX‐ARPES is shown in **Table** **1**, where the physical dopant confinement and electronic thicknesses are stated, respectively. In all cases, we find that arsenic δ‐layers offer a better confinement relative to phosphorus, achieving sub‐nm electronic thicknesses. We attribute this to the smaller diffusion coefficient of arsenic in silicon,^[^ ^52^ ^]^ which, under the same preparation conditions, sustains a more confined δ‐layer than phosphorous.^[^ ^12^ ^]^ Additionally, the δ‐layer thickness was further confirmed by directly fitting the ARPES *k*_z_‐response to the convolution of Lorentzian spectral functions and by taking the Fourier Transform of the probability density function solutions from a Schrödinger–Poisson model of δ‐layers (see Supporting Information). In all instances, a mutual agreement was found.

**Table 1:** Quantifying the δ‐layer confinement. Two independent measures of the δ‐layer confinement using secondary ion mass spectrometry (SIMS) and soft x‐ray ARPES (SX‐ARPES) experiments. All SIMS profiles are shown in the Supporting Information and we note a general agreement with prior measurements in.^[^ ^12^, ^14^, ^15^, ^16^, ^17^ ^]^ For the investigated samples, arsenic δ‐layers consistently yield a better confinement relative to phosphorous. Note that SIMS measures an upper bound on the physical thickness of the δ‐layer dopant distribution, whereas SX‐ARPES measures the electronic thickness; for further details see Supporting Information

δ‐layer species, depth	Physical thickness via SIMS [nm]	Electronic thickness via SX‐ARPES [nm]
As, z = 2 nm	2.01 ± 0.2	0.45 ± 0.04
As, 3 nm	2.22 ± 0.2	0.62 ± 0.10
P, 2 nm	2.24 ± 0.2	0.91 ± 0.21
P, 3 nm	2.86 ± 0.2	1.03 ± 0.35

### 3.3 δ‐Layer Subband Energies and Comparing to Theory

**Figure 4:**
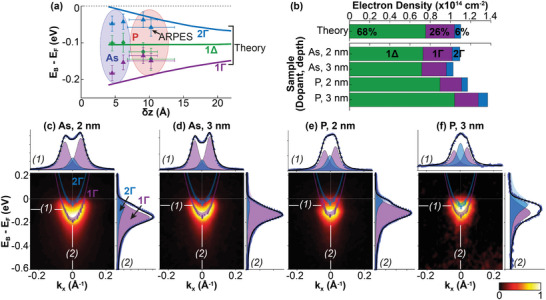
Analysis of the conduction band quantization for phosphorous and arsenic δ‐layers. a) Measured and calculated energies of the δ‐layer subbands. b) Measured and calculated electron density for each δ‐layer subband from the fits acquired in (c–f). For the theory, the percentages denote the occupation of each δ‐layer subband; when the degeneracy is accounted for, each one of the six valleys have an equal occupancy of ≈16.6%. c–f) SX‐ARPES measured Γ band dispersions for 2 and 3 nm phosphorous and arsenic δ‐layers at *h*ν = 380 eV (which corresponds to the centroid of the Γ valley in Figure 3(a)). The purple and blue parabolas are the fits to the ARPES data, showing that the 1 and 2Γ states can be deconvolved. The line profile above each image shows the momentum distribution curves taken at (1); the line profile to the right, labeled (2), shows the corresponding fit to the energy distribution curve.

The analysis of Figures 2 and 3 provide, for each of our samples, a measure of the carrier density and electronic thickness, respectively. These parameters can be used to create an electrostatic model of the δ‐layer (Figure 1a right) that we have used as the basis of self‐consistent Schrödinger–Poisson modeling of the state quantization in *k*_z_ (details of the calculations can be found in Supporting Information). Based on these measured parameters, our calculations show that each of our δ‐layer samples should support 1Γ, 2Γ, and 1Δ states. Additionally, in good agreement with our results, **Figure** **4b** shows that the occupancy of the δ‐layer subbands is also distributed evenly amongst the valleys, in good agreement with our experimental results.^[^ ^42^ ^]^

To further compare these calculations with experiment, we have measured the in‐plane band dispersion and *k*_z_ state quantization directly. Figure 4c,f shows measurements of the band dispersion, *E*_B_(*k*_x_), taken through the centroid of the  + *k*_z_ valley for each of the four samples discussed in Figure 3. We have performed a careful two‐component fit to this data,^[^ ^23^ ^]^ analyzing both iso‐*E*_B_ and iso‐*k*_x_ slices for each data point, as illustrated on the side and top of each panel in Figure 4c–f. Each dataset is best described by two parabolic dispersions, readily interpretable as the 1Γ and 2Γ states expected from the theoretical calculations. A similar analysis of the Δ valley dispersion is provided in the Supporting Information, showing in this case that only a single 1Δ state is observed experimentally. The measured binding energies of these states have been added to the theoretically predicted curves in Figure 4a, and there is good agreement between our calculated and measured band energies in each case.

## 4 Conclusion

We have presented comprehensive SX‐ARPES measurements of dopant δ‐layers in silicon, and revealed that at the high arsenic densities considered, there are three flavors of electrons derived from their confinement along the transverse and longitudinal directions of the conduction band minima of bulk silicon. Our data show that the arsenic δ‐layer samples host the thinnest technological 2D electron liquids ever fabricated in silicon and are close to ideal 2D electron systems with a thickness comparable to the silicon lattice parameter; our thinnest arsenic δ‐layer has an electronic thickness of 0.45 ± 0.04 nm. Moreover, we compared arsenic and phosphorus δ‐layer samples and found that in all cases, the arsenic samples outperformed the phosphorus ones in two‐dimensionality. All our samples are technologically relevant, having been exposed to ambient after their fabrication, demonstrating the remarkable stability of these ultra‐thin, dense δ‐layer systems and the capability of SX‐ARPES to fully characterize their conduction bands directly and non‐destructively. The fact that we can engineer such ultrathin high carrier density liquids represents yet another capability that can be exploited for new nano‐ and quantum‐electronic applications in silicon.

## 5 Experimental Section

### Sample Fabrication

Silicon *n*‐type (10 Ω cm) Si(001) substrates were degassed and flash annealed to ≈1200^○^C under ultra‐high vacuum (< 5 × 10^−10^ mbar). This procedure is known to produce atomically clean surfaces with uniform atomically flat terraces of with widths of 10 to 100s of nanometers.^[^ ^53^ ^]^ The atomically clean and flat surfaces were exposed to a saturation dose of phosphine or arsine, and then annealed at 350^○^C for 2 min to substitutionally incorporate the dopants. The dopant layer was then encapsulated by overgrowing either 2 or 3 nm of silicon using a silicon sublimation source, with a deposition rate of 1 ML/min. During the silicon overgrowth, we controlled the temperature of the sample in three steps to maximize the dopant confinement, following the so‐called locking‐layer procedure^[^ ^12^, ^15^ ^]^: the first 1.3 nm of silicon was grown at room temperature, followed by a rapid thermal anneal at 500^○^C for 15 s and a low‐temperature epitaxial growth at 250^○^C for the remainder of the overgrowth. The samples were then removed from vacuum and exposed to ambient for their transport to the soft X‐ray ARPES facility^[^ ^54^ ^]^ at the Swiss Light Source.

### SX‐ARPES Experiments

The ARPES measurements were performed at the soft X‐ray ARPES facility^[^ ^54^ ^]^ of the ADRESS beamline^[^ ^55^ ^]^ at the Swiss Light Source, PSI, Switzerland. The accessible photon energy range is *h*ν = 300–1600 eV, with a photon flux of up to 10^13^ photons / s / (0.01% BW). To maximize the coherent spectral function (impaired by the thermal atomic motion^[^ ^56^ ^]^), the experiments were performed at a base temperature of 12 K, using circular polarized light. The combined (beamline and analyzer) energy resolution varied from 50 meV at *h*ν = 400 eV, to 90 meV at ≈700 eV. The photoelectron momentum *k*_x_ was directly measured through the emission angle along the analyzer slit, *k*_y_ is varied through the tilt rotation and *k*_z_ is varied through *h*ν. The angular resolution of the ARPES analyzer (PHOIBOS‐150) is 0.1°. Other relevant details of the SX‐ARPES experiments, including experimental geometry can be found in Ref. [54]

## Conflict of Interest

The authors declare no conflict of interest.

## Supporting information



Supporting Information

## Data Availability

The data that support the findings of this study are openly available on Zenodo (zenodo.org) at https://doi.org/10.5281/zenodo.7813819.

## References

[1] T. Ando , A. B. Fowler , F. Stern , Rev. Mod. Phys. 1982, 54, 437.

[2] J. A. Del Alamo , Nature 2011, 479, 317. 22094691 10.1038/nature10677

[3] A. K. Geim , I. V. Grigorieva , Nature 2013, 499, 419. 23887427 10.1038/nature12385

[4] G. Fiori , F. Bonaccorso , G. Iannaccone , T. Palacios , D. Neumaier , A. Seabaugh , S. K. Banerjee , L. Colombo , Nat. Nanotechnol. 2014, 9, 768. 25286272 10.1038/nnano.2014.207

[5] Q. H. Wang , K. Kalantar‐Zadeh , A. Kis , J. N. Coleman , M. S. Strano , Nat. Nanotechnol. 2012, 7, 699. 23132225 10.1038/nnano.2012.193

[6] B. E. Kane , Nature 1998, 393, 133.

[7] C. D. Hill , E. Peretz , S. J. Hile , M. G. House , M. Fuechsle , S. Rogge , M. Y. Simmons , L. C. L. Hollenberg , Sci. Adv. 2015, 1, e1500707. 26601310 10.1126/sciadv.1500707 PMC4646824

[8] M. F. Gonzalez‐Zalba , S. De Franceschi , E. Charbon , T. Meunier , M. Vinet , A. S. Dzurak , Nat. Electron. 2021, 4, 872.

[9] H. P. Zeindl , T. Wegehaupt , I. Eisele , H. Oppolzer , H. Reisinger , G. Tempel , F. Koch , Appl. Phys. Lett. 1987, 50, 1164.

[10] L. Oberbeck , N. J. Curson , M. Y. Simmons , R. Brenner , A. R. Hamilton , S. R. Schofield , R. G. Clark , Appl. Phys. Lett. 2002, 81, 3197.

[11] F. A. Zwanenburg , A. S. Dzurak , A. Morello , M. Y. Simmons , L. C. L. Hollenberg , G. Klimeck , S. Rogge , S. N. Coppersmith , M. A. Eriksson , Rev. Mod. Phys. 2013, 85, 961.

[12] T. J. Z. Stock , O. Warschkow , P. C. Constantinou , J. Li , S. Fearn , E. Crane , E. V. S. Hofmann , A. Kölker , D. R. Mckenzie , S. R. Schofield , N. J. Curson , ACS Nano 2020, 14, 3316. 32142256 10.1021/acsnano.9b08943 PMC7146850

[13] T. Škereň , S. A. Köster , B. Douhard , C. Fleischmann , A. Fuhrer , Nat. Electron. 2020, 3, 524.

[14] X. Wang , J. A. Hagmann , P. Namboodiri , J. Wyrick , K. Li , R. E. Murray , A. Myers , F. Misenkosen , M. D. Stewart , C. A. Richter , R. M. Silver , Nanoscale 2018, 10, 4488. 29459919 10.1039/c7nr07777g PMC11305481

[15] J. G. Keizer , S. Koelling , P. M. Koenraad , M. Y. Simmons , ACS Nano 2015, 9, 12537. 26568129 10.1021/acsnano.5b06299

[16] A. M. Katzenmeyer , T. S. Luk , E. Bussmann , S. Young , E. M. Anderson , M. T. Marshall , J. A. Ohlhausen , P. Kotula , P. Lu , D. M. Campbell , T.‐M. Lu , P. Q. Liu , D. R. Ward , S. Misra , J. Mater. Res. 2020, 35, 2098.

[17] C. M. Polley , W. R. Clarke , J. A. Miwa , G. Scappucci , J. W. Wells , D. L. Jaeger , M. R. Bischof , R. F. Reidy , B. P. Gorman , M. Simmons , ACS Nano 2013, 7, 5499. 23721101 10.1021/nn4016407

[18] D. F. Sullivan , B. E. Kane , P. E. Thompson , Appl. Phys. Lett. 2004, 85, 6362.

[19] G. Matmon , E. Ginossar , B. J. Villis , A. Kölker , T. Lim , H. Solanki , S. R. Schofield , N. J. Curson , J. Li , B. N. Murdin , A. J. Fisher , G. Aeppli , Phys. Rev. B 2018, 97, 17.

[20] F. Koch , A. Zrenner , Mater. Sci. Eng. B 1988, 1, 221.

[21] I. Eisele , Appl. Surf. Sci. 1989, 36, 39.

[22] J. A. Miwa , P. Hofmann , M. Y. Simmons , J. W. Wells , Phys. Rev. Lett. 2013, 110, 136801. 23581353 10.1103/PhysRevLett.110.136801

[23] J. A. Miwa , O. Warschkow , D. J. Carter , N. A. Marks , F. Mazzola , M. Y. Simmons , J. W. Wells , Nano Lett. 2014, 14, 1515. 24571617 10.1021/nl404738j

[24] F. Mazzola , C. M. Polley , J. A. Miwa , M. Y. Simmons , J. W. Wells , Appl. Phys. Lett. 2014, 104, 173108.

[25] F. Mazzola , M. T. Edmonds , K. Høydalsvik , D. J. Carter , N. A. Marks , B. C. C. Cowie , L. Thomsen , J. Miwa , M. Y. Simmons , J. W. Wells , ACS Nano 2014, 8, 10223. 25243326 10.1021/nn5045239

[26] S. Lee , H. Ryu , H. Campbell , L. C. L. Hollenberg , M. Y. Simmons , G. Klimeck , Phys. Rev. B 2011, 84, 205309.

[27] A. J. Holt , S. K. Mahatha , R.‐M. Stan , F. S. Strand , T. Nyborg , D. Curcio , A. K. Schenk , S. P. Cooil , M. Bianchi , J. W. Wells , P. Hofmann , J. A. Miwa , Phys. Rev. B 2020, 101, 121402.

[28] F. Mazzola , C‐Yi Chen , R. Rahman , X.‐G. Zhu , C. M. Polley , T. Balasubramanian , P. D. C. King , P. Hofmann , J. A. Miwa , J. W. Wells , npj Quantum Mater. 2020, 5, 34.

[29] F. Mazzola , J. W. Wells , A. C. Pakpour‐Tabrizi , R. B. Jackman , B. Thiagarajan , Ph. Hofmann , J. A. Miwa , Phys. Rev. Lett. 2018, 120, 046403. 29437461 10.1103/PhysRevLett.120.046403

[30] V. N. Strocov , J. Electron Spectrosc. Relat. Phenom. 2018, 229, 100.

[31] V. N. Strocov , J. Electron Spectrosc. Relat Phenom. 2003, 130, 65.

[32] P. Puschnig , S. Berkebile , A. J. Fleming , G. Koller , K. Emtsev , T. Seyller , J. D. Riley , C. Ambrosch‐Draxl , F. P. Netzer , M. G. Ramsey , Science 2009, 326, 702. 19745118 10.1126/science.1176105

[33] P. Puschnig , M. G. Ramsey , Encycl. Interfacial Chem. 2018, 380, 10.1016/B978-0-12-409547-2.13782-5.

[34] S. Weiß , D. Lüftner , T. Ules , E. M. Reinisch , H. Kaser , A. Gottwald , M. Richter , S. Soubatch , G. Koller , M. G. Ramsey , F. S. Tautz , P. Puschnig , Nat. Commun. 2015, 6, 8287. 26437297 10.1038/ncomms9287 PMC4600719

[35] J. Avila , A. Boury , B. Caja‐Muñoz , C. Chen , S. Lorcy , M. C. Asensio , J. Phys.: Conf. Ser. 2017, 849, 012039.

[36] M. Holler , M. Guizar‐Sicairos , E. H. R. Tsai , R. Dinapoli , E. Müller , O. Bunk , J. Raabe , G. Aeppli , Nature 2017, 543, 402. 28300088 10.1038/nature21698

[37] M. Holler , M. Odstrcil , M. Guizar‐Sicairos , M. Lebugle , E. Müller , S. Finizio , G. Tinti , C. David , J. Zusman , W. Unglaub , O. Bunk , J. Raabe , A. F. J. Levi , G. Aeppli , Nat. Electron. 2019, 2, 464.

[38] F. Joucken , J. Avila , Z. Ge , E. A. Quezada‐Lopez , H. Yi , R. Le Goff , E. Baudin , J. L. Davenport , K. Watanabe , T. Taniguchi , M. C. Asensio , J. Velasco , Nano Lett. 2019, 19, 2682. 30888827 10.1021/acs.nanolett.9b00649

[39] P. Majchrzak , R. Muzzio , A. J. H. Jones , D. Curcio , K. Volckaert , D. Biswas , J. Gobbo , S. Singh , J. T. Robinson , K. Watanabe , T. Taniguchi , T. K. Kim , C. Cacho , J. A. Miwa , P. Hofmann , J. Katoch , S. Ulstrup , Small Sci. 2021, 1, 2000075. 10.1002/smsc.202000075 PMC11936027 40212713

[40] G. Gramse , A. Kölker , T. Škereň , T. J. Z. Stock , G. Aeppli , F. Kienberger , A. Fuhrer , N. J. Curson , Nat. Electron. 2020, 3, 531.

[41] F. Herman , Proc. IRE 1955, 43, 1703.

[42] D. W. Drumm , L. C. L. Hollenberg , M. Y. Simmons , M. Friesen , Phys. Rev. B 2012, 85, 155419.

[43] D. J. Carter , O. Warschkow , N. A. Marks , D. R. Mckenzie , Phys. Rev. B 2009, 79, 033204.

[44] D. J. Carter , N. A. Marks , O. Warschkow , D. R. Mckenzie , Nanotechnology 2011, 22, 065701. 21212477 10.1088/0957-4484/22/6/065701

[45] D. J. Carter , O. Warschkow , N. A. Marks , D. R. Mckenzie , Phys. Rev. B 2013, 87, 045204.

[46] S. G. Louie , P. Thiry , R. Pinchaux , Y. Pétroff , D. Chandesris , J. Lecante , Phys. Rev. Lett. 1980, 44, 549.

[47] S. D. Kevan , N. G. Stoffel , N. V. Smith , Phys. Rev. B 1985, 31, 3348. 10.1103/physrevb.31.3348 9936220

[48] D.‐S. Lin , T.‐S. Ku , T.‐J. Sheu , Surf. Sci. 1999, 424, 7.

[49] M. A. Berding , A. Sher , M. Van Schilfgaarde , P. M. Rousseau , W. E. Spicer , Appl. Phys. Lett. 1998, 72, 1492.

[50] S. Duguay , F. Vurpillot , T. Philippe , E. Cadel , R. Lardé , B. Deconihout , G. Servanton , R. Pantel , J. Appl. Phys. 2009, 106, 106102.

[51] G. Dresselhaus , A. F. Kip , C. Kittel , Phys. Rev. 1955, 98, 368.

[52] P. M. Fahey , P. B. Griffin , J. D. Plummer , Rev. Mod. Phys. 1989, 61, 289.

[53] B. S. Swartzentruber , Y.‐W. Mo , M. B. Webb , M. G. Lagally , J. Vac. Sci. Technol., A 1989, 7, 2901.

[54] V. N. Strocov , M. Kobayashi , X. Wang , L. L. Lev , J. Krempasky , V. V. Rogalev , T. Schmitt , C. Cancellieri , M. L. Reinle‐Schmitt , Synchrotron Radiat. News 2014, 27, 31.

[55] V. N. Strocov , T. Schmitt , U. Flechsig , T. Schmidt , A. Imhof , Q. Chen , J. Raabe , R. Betemps , D. Zimoch , J. Krempasky , X. Wang , M. Grioni , A. Piazzalunga , L. Patthey , J. Synchrotron Radiat. 2010, 17, 631. 20724785 10.1107/S0909049510019862 PMC2927903

[56] J. Braun , J. Minár , S. Mankovsky , V. N. Strocov , N. B. Brookes , L. Plucinski , C. M. Schneider , C. S. Fadley , H. Ebert , Phys. Rev. B 2013, 88, 205409.

