# Supplementary material for: Momentum‐Space Imaging of Ultra‐Thin Electron Liquids in δ‐Doped Silicon
Source: Adv Sci (Weinh). 2023 Jul 19;10(27):2302101. doi: 10.1002/advs.202302101 (PMC10520640; doi:10.1002/advs.202302101)
Supplement: Supplementary file 1 — Supporting Information [file ADVS-10-2302101-s001.pdf]

ADVANCED SCIENCE
Open Access

Supporting Information

for *Adv. Sci.*, DOI 10.1002/advs.202302101

Momentum-Space Imaging of Ultra-Thin Electron Liquids in δ-Doped Silicon

Procopios Constantinou, Taylor J. Z. Stock, Eleanor Crane, Alexander Kölker, Marcel van Loon, Juerong Li, Sarah Fearn, Henric Bornemann, Nicolò D'Anna, Andrew J. Fisher, Vladimir N. Strocov, Gabriel Aeppli, Neil J. Curson and Steven R. Schofield**

Supporting Information: Momentum space imaging of ultra-thin electron liquids in δ-doped silicon

Procopios Constantinou*[1,2,5], Taylor J. Z. Stock[1,9], Eleanor Crane[1,9], Alexander Kölker[1,9], Marcel van Loon[1,2], Juerong Li[3], Sarah Fearn[1,4], Henric Bornemann[1,2], Nicolò D'Anna[5], Andrew J. Fisher[1,2], Vladimir N. Strocov[5], Gabriel Aeppli[5,6,7,8], Neil J. Curson[1,9], Steven R. Schofield†[1,2]

[1] London Centre for Nanotechnology, University College London, WC1H 0AH, London, UK

[2] Department of Physics and Astronomy, University College London, WC1E 6BT, London, UK

[3] Advanced Technology Institute, University of Surrey, Guildford GU2 7XH, UK

[4] Department of Materials, Imperial College of London, London SW7 2AZ, UK

[5] Paul Scherrer Institute, 5232 Villigen, Switzerland

[6] Institute of Physics, Ecole Polytechnique Fédérale de Lausanne (EPFL), 1015 Lausanne

[7] Department of Physics, ETH Zürich, 8093 Zürich

[8] Quantum Center, Eidgenössische Technische Hochschule Zurich (ETHZ), 8093 Zurich, Switzerland

[9] Department of Electronic and Electrical Engineering, University College London, London WC1E 7JE, UK

* procopios.constantinou@psi.ch

† s.schofield@ucl.ac.uk

(Dated: Sunday, 09 July 2023)

Supplementary Information Contents

(A) SIMS depth profiling of δ-layer samples 2

(B) Hall measurements of δ-layer samples 3

(C) ARPES of samples with and without a δ-layer along XW 3

(D) Quantifying the δ-layer carrier density 4

(E) Quantifying the δ-layer thickness by fitting the k_z response 4

(F) Γ- and Δ-band fitting procedures 5

(G) Self-consistent Schrödinger-Poisson model of δ-layers 6

Supplementary Information References 9

Supplementary Information Figures 11

(A) SIMS depth profiling of δ-layer samples

Time-of-flight secondary ion mass spectrometry (ToF-SIMS) measurements were made using an IONTOF ToF.SIMS5-Qtac100 system at the Surface Analysis Facility at Imperial College London. The base pressure was below 10^{-10} mbar, with a mass resolution $m/\Delta m = 10{,}000$. The ToF-SIMS system was operated in static mode, with a 25 keV Bi^+ primary ion beam in high current bunch mode (HCBM). For depth profiling, a 250 eV, 20 nA Cs^+ sputter ion beam was used, with an impact angle at 45° to the sample surface, with an estimated sputter rate of (0.03 ± 0.02) nm/s. These settings were found to maximise the depth resolution and relative sensitivity factor, whilst minimising the atomic mixing and straggle depth [S1], [S2]. Depth profiles were made over a 300 μm × 300 μm sputter crater, where the analytical region was gated to the central 50 μm × 50 μm to minimise the artefacts of the non-uniform edges of the sputter crater. The depth of the SIMS craters was measured using a Zygo NewView 200 3D optical interferometer; this determines a uniform sputter rate for each SIMS measurement. To calibrate the secondary ion intensity to a volumetric density, a standard δ-layer sample with a known δ-layer density was used as reference.

Since the secondary ion yield for a given dopant species depends on the matrix it is embedded in, a normalisation procedure is required for a complete interpretation of the SIMS data. In the literature, accurate quantification has been studied for ultra-shallow dopant layers buried in a SiO_2 and Si stack, where either the SiO_2 or Si secondary ion signals are used for normalisation [S3]. Here, we normalise the dopant signal to the sum of the SiO_2 and Si signals, since the δ-layers lie within a convoluted SiO_2 and Si matrix.

Figure S1 shows the SIMS depth profiles of identically prepared phosphorous (red) and arsenic (blue) δ-layers buried 2 and 3 nm below the Si(001) surface. In comparing Figure S1(b,c) and Figure S1(d,e), we find that an arsenic δ-layer yields a better dopant confinement in the direction perpendicular to the δ-layer at comparative depths: (i) For the 2 nm deep arsenic and phosphorous δ-layer, a full width at half maximum (FWHM) of (2.0 ± 0.2) nm and (2.2 ± 0.2) nm is measured respectively; (ii) For the 3 nm deep arsenic and phosphorous δ-layer, a FWHM of (2.2 ± 0.2) nm and (2.9 ± 0.2) nm is measured, respectively. We attribute this to the smaller diffusion constant of arsenic in silicon [S4], [S5], which allows arsenic to remain more confined, relative to phosphorous, for equivalent thermal budgets of sample growth. It is important to add that the SIMS measure of the FWHM is an upper-bound estimate at best, as the depth profiles are inherently broadened dues to instrumental parameters, ion beam interactions and element-specific detections [S6], [S7]. Thus, we expect the actual δ-layers to be more confined than the results in Figure S1.

By integrating the area beneath the dopant signal of the SIMS depth profiles, the δ-layer sheet density can be determined. Here, the density is defined in terms of a monolayer (ML), which conveniently translates to the fractional coverage of dopants on the initial Si(001) surface, where $1\ \mathrm{ML} = 6.78 \times 10^{14}\ \mathrm{cm}^2$. For phosphorous and arsenic, we find a δ-layer density of (0.36 ± 0.04) ML and (0.27 ± 0.03) ML respectively; or $(2.5 \pm 0.3) \times 10^{14}\mathrm{cm}^{-2}$ and $(1.8 \pm 0.2) \times 10^{14}\mathrm{cm}^{-2}$, respectively. This difference in the saturation density is expected due to the differences in the dissociation chemistry of the dopant precursors [S8], [S9].

(B) Hall measurements of δ-layer samples

Eight-terminal Hall bars were fabricated from the phosphorous and arsenic δ-layer samples after the SX-ARPES experiments. Figure S2(a) is a schematic of the final, etched Hall-bar mesa. After cleanroom processing, the samples were then electrically characterised at ≈ 10 K with Hall measurements, whose results are shown in Figure S2(b). By fitting to these data, we can quantify the carrier concentration and mobility for each sample. This is shown in Table S1. Note, we only show the data for the 3 nm deep arsenic δ-layer, as the 2 nm arsenic δ-layer sample did not display any transport due to open-circuit contacts; we attribute this to the potential failure of the etching process during cleanroom processing.

We find that the carrier concentration determined from the Hall experiments in Figure S2 is smaller than the δ-layer densities extracted from SIMS. This is expected, as not all incorporated donors provide electrons and there are several mechanisms for dose loss; deactivated donors in interstitials, vacancies or due to surface oxidation. Additionally, it is important to state that the samples were HF etched prior to the deposition of the Al contacts shown in Figure S2(a), which is required to achieve ohmic contact to the δ-layer. This inevitably brings the δ-layer closer to the surface and leads to another regrowth of the surface oxide; hence, a further dose loss of the δ-layers is expected relative to the initial SX-ARPES measurements. Nevertheless, the carrier concentrations remain well above the metal-insulator transition. In particular, since arsenic is known to suffer from clustering at high concentrations [S10], [S11], this further results in incomplete activation, and we attribute this to the smaller carrier concentration of the arsenic δ-layers compared to phosphorous in Table S1.

(C) ARPES of samples with and without a δ-layer along XW

By aligning the ARPES measurement plane to coincide with the ΓX symmetry plane and tuning the photon energy to 380 eV, the $E_B(k_x)$ band dispersion along the XW symmetry line is mapped. Figure S3 shows a comparison of the ARPES data for a Si(001) sample without a δ-layer (Figure S3(a)), with a 2 and 3 nm deep arsenic (Figure S3(b)) and phosphorous (Figure S3(c)) δ-layer. The data immediately show that the sample without a δ-layer has no occupation of the conduction band. This is expected as the lack of any doping implies that the Fermi-level resides within the bandgap. However, both types of δ-layer samples show a bright conduction band pocket at the bulk X-point, which is a clear signature of the metallic nature of these δ-layers. A crucial point is that the photon energy is identical for all the ARPES data shown in Figure S3, so the inelastic mean free path (IMFP) of the emitted photoelectrons is constant. Thus, for a δ-layer buried deeper beneath the surface, we would expect it to yield a weaker spectral intensity, due to the sub-surface nature of the δ-layer. This is observed in Figure S3(b) and Figure S3(c), where the 3 nm deep δ-layers both show a diminished intensity relative to the 2 nm deep δ-layer. This provides a clear indication that the occupied conduction band state originates from the sub-surface δ-layer.

(D) Quantifying the δ-layer carrier density

We discuss here the procedure used to quantify the carrier concentration from the ARPES Fermi surface. All the relevant ARPES data are shown in Figure S4 for both the arsenic and phosphorous δ-layer samples, where k_x-k_y slices are taken through the conduction valleys. Note, in the bottom panel of Figure S4(d), the $+k_x$ valley does not appear as expected and we attribute this to a slight rotational misalignment during the acquisition of the ARPES data.

The total area enclosed by each valley is determined consistently by thresholding the ARPES data at two iso-contour limits; values at which the maximum intensity drops to 25% (upper bound estimate of the enclosed area) and 50% (lower bound estimate of the enclosed area). These two limits are shown in Figure S4(a-d) as shaded regions around each valley; coloured purple for the $+k_z$ valley (middle panel) and green for the in-plane ($\pm k_x$,$\pm k_y$) valleys (bottom panel). An estimate of the carrier concentration, n_e, is then determined by applying *Luttinger's Theorem* [S12], which states that the total area enclosed by the Fermi surface is directly proportional to the number density of electrons. A simple expression for this can be derived from the free-electron (Sommerfeld) model, where:

$$n_e = 2\frac{1}{(2\pi)^j}V_{kj}\,. \tag{5}$$

Here, V_{kj} is the j-dimensional volume of k-space, where in the case for the two-dimensional δ-layer system, $j = 2$. The result of applying Equation (5) is shown in the upper-panel of Figure S4(a-d), which shows a breakdown of the carrier concentration for each one of the six valleys. We find that within the experimental uncertainty, there is an equal occupancy amongst each valley, where the sum yields the total carrier concentration of each δ-layer sample. Since the $n = 2$ state of the Γ-valley cannot be clearly resolved within the Fermi-surfaces shown in the middle panel of Figure S4(a-d), the contribution of the 2Γ state is extracted from the fits to Figure S6(a-d). A free-electron like (or circular) Fermi-surface is assumed. In doing so, the carrier concentration estimate slightly increases by 10% and the total carrier concentration for all δ-layer samples is found to lie within $(0.88 \pm 0.10) \times 10^{14}$ cm^{-2}.

Alternatively, it is known that a more accurate method to determine the total area enclosed by the Fermi surface is to extract the maximum gradient of the energy integrated photoemission intensity from the experimental ARPES data [S13]. This so-called gradient method is based on the theoretical many-body definition of the Fermi surface and this analysis was implemented for the 2 nm arsenic δ-layer sample, using the data in Figure S4(a). In doing so, the total carrier concentration was found to be approximately the same; ~15% higher.

(E) Quantifying the δ-layer thickness by fitting the k_z response

Here, we compare the two methods of quantifying the δ-layer confinement (δz) discussed in the main manuscript and show that they are in mutual agreement (Figure S5(g)). The first method is to apply Equation (1) from the main manuscript, where δz is the reciprocal of the difference in the longitudinal span of the Γ- and Δ-valleys (Figure 3). The second method relies on fitting the k_z response of the data to the convolution of a pair of mean free path (MFP) broadened Lorentzian's, to another Lorentzian whose width is proportional to δz^{-1} (Figure

S5(a)). A Lorentzian curve shape is used since the Fourier Transform of an exponentially decaying wavefunction is Lorentzian, and a pair of curves due to the $\pm k_z$ momenta of the initial states that are in superposition and form a standing wave perpendicular to the δ-layer. The TPP-2M formalism for silicon [S14] is used to estimate the photoelectron MFP, λ_e, via the incident photon energy, $h\nu$; we find λ_e spans 1.2 – 2.0 nm for $h\nu$ between 380 – 820 eV. Furthermore, we neglect the contribution from the spectrometer energy resolution, which translates into a momentum broadening of $\Delta k \approx 0.02\text{Å}^{-1}$ about the same $h\nu$ range. The initial position of the MFP-broadened peaks is estimated by finding the corresponding Fermi wave-vector, k_F, of the Δ-valleys in k_x-k_y. A least-squares approach is employed to find the optimal value of δz required to fit the k_z response of both the Γ- and Δ-valleys.

Horizontal (k_z) and vertical (k_x) cuts of the Fermi-surface are shown in Figure S5(a), with the 1Γ and 2Γ states being identified. We observe that the first quantum well state (1Γ) brackets the second quantum well state (2Γ), and that the intensity of the k_z line-profile across the Γ-valley is dominated by the 1Γ contribution. Thus, when fitting the k_z-response of the data, we found near identical results for k_x intervals that are either localised around 1Γ, or integrated across the whole Γ-valley. Additionally, by taking the Fourier Transform of the probability density function solutions from the Schrödinger-Poisson model of δ-layers derived in Figure S7 (discussed further in Section G), we find that they also provide a good agreement to the k_z ARPES spectral response. This is identically shown for the Δ-valley in Figure S5(b), where the 1Δ singlet state can be identified. Furthermore, we again find that the Fourier Transform coincides with theoretical expectations, which also appears more localised along k_z; the sharper k_z-response is associated with the 1Δ wave function being broader in real-space (see Figure S7).

Figure S5(c-f) shows the best estimate of δz using the model fit approach outlined in Figure S5(a-b) and discussed above. We find that arsenic δ-layers yield a better confinement relative to phosphorous and that the fits to both the Γ- and Δ-valleys yield consistent values of δz. Furthermore, Figure S5(g) confirms that either of the approaches discussed above can be used to estimate δz, as the values are in mutual agreement. This works because the convolution of Lorentzian's yields a Lorentzian whose width is the arithmetic sum of the component widths.

(F) Γ- and Δ-band fitting procedures

The procedures used to fit the ARPES data shown in Figure 4 of the main manuscript are discussed here. A summary of the Γ-band fits are shown in Figure S6(a-d) and the Δ-band fits are shown in Figure S6(e-h), for all δ-layer samples. Each panel shows the ARPES data, overlaid with the best fit parabolic bands, where the number in brackets identifies the type of cut that is taken through the ARPES data. There are two cuts used in the fitting of the ARPES data; (i) momentum distribution curves (MDCs) or horizontal cuts (shown above the ARPES data) and (ii) energy distribution curves (EDCs) or vertical cuts (shown to the right of the ARPES data). We find that the Γ-band fits can be deconvolved into two discrete states, denoted as 1Γ and 2Γ, whereas the Δ-band fits are deconvolved into a single state, 1Δ.

Initially when trying to fit the Γ-bands consistently with a single state, the middle regions of the MDCs in Figure S6(a-d) (labelled as (1)-(3)) were found to be significantly underestimated, with no satisfactory fit being achieved. It was also found that when using pure Gaussian functions, the MDC cuts would not fit well due to the extended tails that are formed by each of the peaks. The best fits made for each sample are shown in Figure S6(a-d), where the fits are comprised of multiple peaks that follow a parabolic path in k_x, derived from two discrete sub-bands, which we denote as 1Γ and 2Γ. A least-square fitting algorithm is employed in MATLAB that consists of fitting a series of Voigt functions, equally weighted in terms of its Gaussian and Lorentzian parts, that each follow an initially parabolic path in $E_B(k_x)$ until a satisfactory fit converges through each MDC cut. This form was chosen as it combined the experimental resolution and intrinsic broadening effects, whilst optimally fitting the data. The fitting algorithm starts at an MDC cut taken at -0.2 eV, and progressively moves upwards through the Γ-state fitting up to four Voigt functions (for $n = 2$ states) until it hits the Fermi-level, after which it terminates. The results of this are overlaid on the ARPES images of Figure S6(a-d). To ensure consistency, all fits were performed using similar constraints. The FWHM is constrained to $(0.06 \pm 0.03)\text{Å}^{-1}$ and the best fit parabola for all δ-layer samples yielded an effective mass of $(0.12 \pm 0.03)m_e$, which lies close to the expected value of $0.19m_e$ [S15]. Furthermore, the EDC cut through the centre of mass of the Γ-band in Figure S6(a-d) (labelled as (4)) also shows a satisfactory fit when using two components. It is important to note that the model curve used to fit the EDC case is more complicated; it is the convolution of a Voigt function with a Gaussian broadened Fermi-Dirac distribution (the Gaussian broadening is a consequence of the 60 meV energy resolution). Therefore, the EDC curve fits appear asymmetrical and drop to zero near $E_B = E_F$.

For the Δ-band fits shown in Figure S6(e-h), the fits are simpler as a single state was found to yield satisfactory results. However, due to the higher effective mass of the Δ-band, its much smaller curvature makes it more difficult to track the peak locations, which is why the best fit parabola has a larger uncertainty, relative to the Γ-band fits. Because of this, the fits were made to each EDC cut from left to right, using the same algorithm discussed above. For the arsenic δ-layer samples in Figure S6(e-f), the peak position appears to be constant when fitting the EDCs, however, this is due to the base of the Δ-band being very near the Fermi-edge and its very low curvature at the base of the parabola makes it seem flat. For phosphorous δ-layer samples in Figure S6(g-h), the parabolic minimum is further away from the Fermi-edge and so the slight curvature of the Δ-band becomes apparent. To ensure consistency, all fits were performed using similar constraints. The FWHM of the EDC fits were constrained to (0.16 ± 0.04) eV and the best fit parabola for all δ-layer samples yielded an effective mass of $(0.90 \pm 0.05)m_e$, which lies close to the expected value of $0.98m_e$ [S15].

(G) Self-consistent Schrödinger-Poisson model of δ-layers

The theoretical model being compared to the experimental data in Figure 4 of the main manuscript is outlined here. The model employs an effective mass approximation [S16] and accounts for dopant segregation in realistic δ-layers [S17]. Given the latter, the model also

neglects the inter-valley coupling, which becomes vanishingly small for δ-layers wider than 0.2 nm [S17].

In highly doped δ-layers, a strong electrostatic potential is induced that confines electrons to the plane. The high concentration of donor electrons leads to a substantial overlap of their wavefunction and screening of the attractive cores. As a result, the local disorder is smoothed out. It is therefore convenient to ignore the placement of individual atoms and describe the layer by a constant average charge density. The system now exhibits planar symmetries, where the electrostatic potential only varies in the plane-perpendicular direction. The infinite δ-layer is thus described by one-dimensional Schrödinger-like equations:

$$\left(-\frac{\hbar^2}{2m_{\parallel}}\frac{d^2}{dz^2}+V(z)\right)F_\Gamma(z)=\epsilon_\Gamma F_\Gamma(z)\,, \tag{7}$$

$$\left(-\frac{\hbar^2}{2m_{\perp}}\frac{d^2}{dz^2}+V(z)\right)F_\Delta(z)=\epsilon_\Delta F_\Delta(z)\,. \tag{8}$$

Here, $m_{\parallel} \approx 0.98m_e$ corresponds to the longitudinal anisotropic electron mass in silicon and $m_{\perp} \approx 0.19m_e$ the transverse effective mass given in terms of the bare electron mass m_e. Note that the equations are not given in terms of the full electronic wavefunction $\psi(\mathbf{r}) = F(\mathbf{r})\psi(\mathbf{k},\mathbf{r})$, but instead of envelope functions F_i. The complete solutions of the wave equations include rapid Bloch oscillations that can be omitted in the effective mass approximation. The Schrödinger-like equations are re-written as systems of first order linear equations. The eigenvalues ϵ_i and envelope functions are computed using a root-finding Schrödinger solver and the second order Runge-Kutta method. Note that the model neglects inter-valley coupling. This is appropriate for the current samples, as the valley splitting of the 1Γ band becomes vanishingly small for δ-layers wider than 0.2 nm [S17].

Assuming the electron wavefunctions are separable, the plane-parallel solutions to the wave equation correspond to a two-dimensional electron gas (2DEG). Each of the quantised bands from the plane-perpendicular envelope functions can thus be occupied by electrons obeying the state filling rules of a 2DEG. Taking the two- and four-fold degeneracy of the bands into account, the following set of equations are obtained:

$$\begin{aligned} 2(\epsilon_F-\epsilon_{n\Gamma})D_\Gamma(\epsilon)&=N_{n\Gamma}\,,\\ 4(\epsilon_F-\epsilon_{n\Delta})D_\Delta(\epsilon)&=N_{n\Delta}\,, \end{aligned} \tag{9}$$

where $D_\Gamma(\epsilon)=m_\perp/\pi\hbar^2$ and $D_\Delta(\epsilon)=\sqrt{m_\parallel m_\perp}/\pi\hbar^2$ are the density of states per unit area of the corresponding bands. The Fermi energy is found by the charge neutrality condition, i.e. the total number of electrons $N_e=\sum_i N_i$ equals the total number of donors N, and by finding a self-consistent solution for:

$$\epsilon_F=\frac{N+2D_\Gamma\sum_j^{M_\Gamma}\epsilon_j^\Gamma+4D_\Delta\sum_k^{M_\Delta}\epsilon_k^\Delta}{2M_\Gamma D_\Gamma+4M_\Delta D_\Delta}\,, \tag{10}$$

where M_i are the number of bands below the Fermi energy. The electrons are distributed to the different bands according to Equation (9).

The potential $V(z)=V_H[n(z)]+V_{XC}[n(z)]$ is obtained from the resulting electron carrier distribution. The exchange-correlation potential V_{XC} is computed with the XC functional by *Ceperley and Adler* and by employing scaled atomic units in the parametrization by *Perdew and Zunger* [S18]. The Hartree potential V_H is obtained from Poisson's equation by applying

the finite difference method. The method maps the problem onto a grid, such that the second derivative of the potential becomes:

$$(\partial_z^2 V)_i \approx \frac{V_{i-1} - 2V_i + V_{i+1}}{dz^2}, \tag{11}$$

where i are the indices of the grid. By adopting the matrix representation and imposing the boundary conditions $\lim_{z\to\pm\infty} V(z) = 0$, the Poisson equation takes the form:

$$\underbrace{\frac{1}{dz^2}\begin{pmatrix} -2 & 1 & 0 & \dots & 0 \\ 1 & -2 & 1 & \dots & 0 \\ 0 & \ddots & \ddots & \ddots & \vdots \\ \vdots & \ddots & 1 & -2 & 1 \\ 0 & \dots & 0 & 1 & -2 \end{pmatrix}}_{=M} \cdot \begin{pmatrix} V_0 \\ V_1 \\ \vdots \\ V_{b_z-1} \\ V_{b_z} \end{pmatrix} = \frac{e}{\epsilon_r \epsilon_0} \begin{pmatrix} n_0 \\ n_1 \\ \vdots \\ n_{b_z-1} \\ n_{b_z} \end{pmatrix}, \tag{12}$$

where b_z is the number of discrete points centred around $z = 0$. Multiplication with the inverse matrix M^{-1} yields the Hartree potential.

Unlike the effective mass approximation in Ref. [S16], the donor atoms are not confined to one doping layer but are subject to a sharp Gaussian segregation profile, in line with Ref. [S17]. Adding up the opposing electrostatic potentials from dopants and electrons and the exchange-correlation contribution gives the total potential of the system $\tilde{V}(z)$. The calculation is repeated with $V(z) = \tilde{V}(z)$ until self-consistency is reached. It occurs when $\int [V_{\alpha+1}(z) - V_\alpha(z)] dz \approx 0$ is satisfied, where α and $\alpha + 1$ refer to successive iterations.

Figure S7 summarises the results obtained when using the self-consistent Schrödinger-Poisson model of δ-layers. The wave-functions of the occupied Γ- and Δ-band states are shown in Figure S7(a-b), along with their corresponding carrier concentrations. We find an equal occupancy amongst the Γ- and Δ-bands for metallic δ-layers, whose density is of the order 10^{14}cm^{-2}. Figure S7(c) also shows the evolution of the energy minima of each sub-band as a function of the FWHM of the δ-layer; as the δ-layer becomes broader, the effect of energy quantisation becomes smaller.

Supplementary Information References

[S1] S. Hofmann, “Ultimate depth resolution and profile reconstruction in sputter profiling with AES and SIMS,” *Surface and Interface Analysis*, vol. 30, no. 1, pp. 228–236, 2000, doi: 10.1002/1096-9918(200008)30:1<228::AID-SIA821>3.0.CO;2-E.

[S2] A. Merkulov, P. Peres, S. Choi, F. Desse, and M. Schuhmacher, “Advanced SIMS quantification in the first few nm of B, P and As ultrashallow implants,” *Surface and Interface Analysis*, vol. 43, no. 1–2, pp. 522–524, 2011, doi: 10.1002/sia.3459.

[S3] M. Barozzi, D. Giubertoni, M. Anderle, and M. Bersani, “Arsenic shallow depth profiling: accurate quantification in SiO2/Si stack,” *Appl Surf Sci*, vol. 231–232, pp. 632–635, Jun. 2004, doi: 10.1016/j.apsusc.2004.03.127.

[S4] C. S. Fuller and J. A. Ditzenberger, “Diffusion of Donor and Acceptor Elements in Silicon,” *J Appl Phys*, vol. 27, no. 5, pp. 544–553, May 1956, doi: 10.1063/1.1722419.

[S5] P. M. Fahey, P. B. Griffin, and J. D. Plummer, “Point defects and dopant diffusion in silicon,” *Rev Mod Phys*, vol. 61, no. 2, pp. 289–384, Apr. 1989, doi: 10.1103/RevModPhys.61.289.

[S6] S. Hofmann, “Approaching the limits of high resolution depth profiling,” *Appl Surf Sci*, vol. 70–71, no. PART 1, pp. 9–19, Jun. 1993, doi: 10.1016/0169-4332(93)90389-S.

[S7] H. Werner and P. Boudewijn, “A comparison of SIMS with other techniques based on ion-beam solid interactions,” *Vacuum*, vol. 34, no. 1–2, pp. 83–101, Jan. 1984, doi: 10.1016/0042-207X(84)90111-8.

[S8] T. J. Z. Stock *et al.*, “Atomic-Scale Patterning of Arsenic in Silicon by Scanning Tunneling Microscopy,” *ACS Nano*, vol. 14, no. 3, pp. 3316–3327, Mar. 2020, doi: 10.1021/acsnano.9b08943.

[S9] D. S. Lin, T. S. Ku, and T. J. Sheu, “Thermal reactions of phosphine with Si(100): A combined photoemission and scanning-tunneling-microscopy study,” *Surf Sci*, vol. 424, no. 1, pp. 7–18, 1999, doi: 10.1016/S0039-6028(98)00943-1.

[S10] M. A. Berding, A. Sher, M. van Schilfgaarde, P. M. Rousseau, and W. E. Spicer, “Deactivation in heavily arsenic-doped silicon,” *Appl Phys Lett*, vol. 72, no. 12, pp. 1492–1494, Mar. 1998, doi: 10.1063/1.121036.

[S11] S. Duguay *et al.*, “Evidence of atomic-scale arsenic clustering in highly doped silicon,” *J Appl Phys*, vol. 106, no. 10, p. 106102, Nov. 2009, doi: 10.1063/1.3257178.

[S12] T. D. Stanescu, P. Phillips, and T.-P. Choy, “Theory of the Luttinger surface in doped Mott insulators,” *Phys Rev B*, vol. 75, no. 10, p. 104503, Mar. 2007, doi: 10.1103/PhysRevB.75.104503.

[S13] Th. Straub *et al.*, “Many-body definition of a Fermi surface: Application to angle-resolved photoemission,” *Phys Rev B*, vol. 55, no. 20, pp. 13473–13478, May 1997, doi: 10.1103/PhysRevB.55.13473.

[S14] S. Tanuma, C. J. Powell, and D. R. Penn, “Calculations of electron inelastic mean free paths. IX. Data for 41 elemental solids over the 50 eV to 30 keV range,” *Surface and Interface Analysis*, vol. 43, no. 3, pp. 689–713, Mar. 2011, doi: 10.1002/sia.3522.

[S15] G. Dresselhaus, A. F. Kip, and C. Kittel, “Cyclotron Resonance of Electrons and Holes in Silicon and Germanium Crystals,” *Physical Review*, vol. 98, no. 2, pp. 368–384, Apr. 1955, doi: 10.1103/PhysRev.98.368.

[S16] D. W. Drumm, L. C. L. Hollenberg, M. Y. Simmons, and M. Friesen, "Effective mass theory of monolayer δ doping in the high-density limit," *Phys Rev B*, vol. 85, no. 15, p. 155419, Apr. 2012, doi: 10.1103/PhysRevB.85.155419.

[S17] S. Lee, H. Ryu, H. Campbell, L. C. L. Hollenberg, M. Y. Simmons, and G. Klimeck, "Electronic structure of realistically extended atomistically resolved disordered Si:P δ-doped layers," *Phys Rev B*, vol. 84, no. 20, p. 205309, Nov. 2011, doi: 10.1103/PhysRevB.84.205309.

[S18] J. P. Perdew and A. Zunger, "Self-interaction correction to density-functional approximations for many-electron systems," *Phys Rev B*, vol. 23, no. 10, pp. 5048–5079, 1981, doi: 10.1103/PhysRevB.23.5048.

Supplementary Information Figures

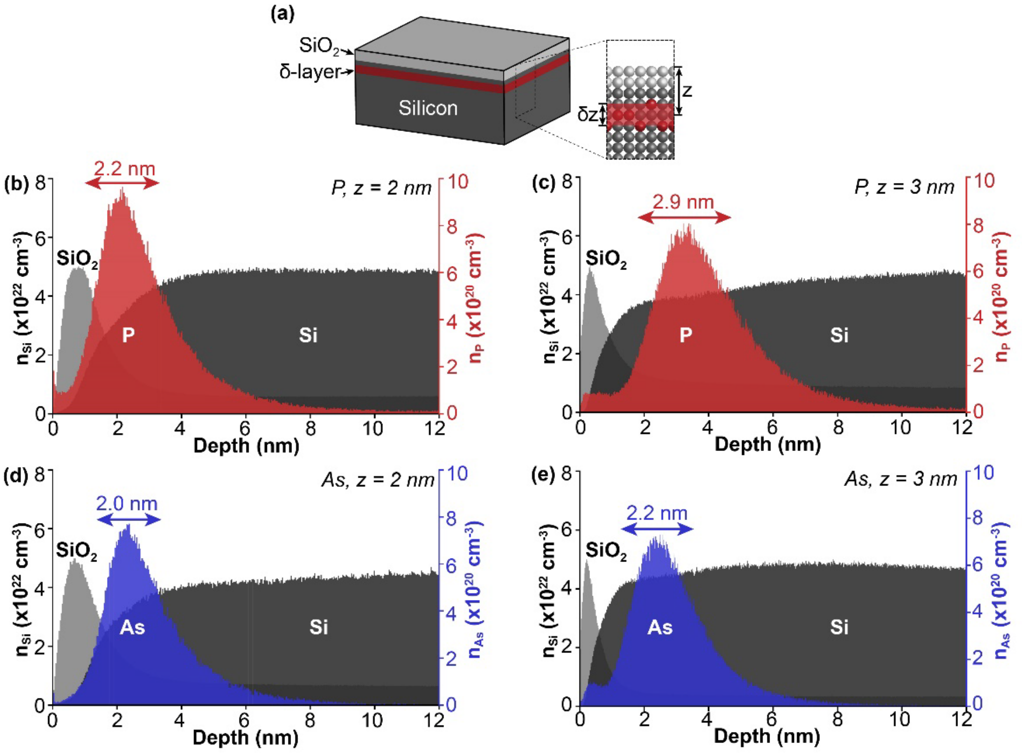

Figure S1: SIMS depth profiles of ultra-shallow *δ*-layer samples. (a) Schematic representation of the samples prepared; z is the silicon overgrowth thickness, noting surface oxidation will have occurred, and δz is the thickness of the δ-layer. (b,c) Secondary ion mass spectrometry (SIMS) of phosphorous δ-layer samples fabricated with (b) 2 nm, (c) 3 nm of silicon overgrowth. (d,e) SIMS of the arsenic δ-layer samples fabricated with (d) 2 nm, (e) 3 nm of silicon overgrowth. The Si(dark grey), SiO_2 (light grey), P (red) and As (blue) signals are shown. The Si signal is derived from the Si_3 fragmentation species. The data are calibrated to known fluence standards.

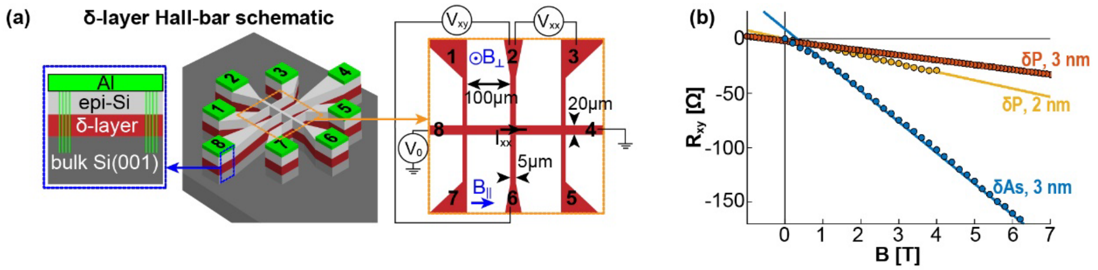

Figure S2: Summary of the magnetotransport measurements of δ-layer samples. (a) Schematic of the Hall-bar mesa created after cleanroom processing. A cross-section of the δ-layer device stack is shown to the left, contacted with aluminium vias. A zoom in of the Hall-bar geometry is shown to the right, where the orientation of the magnetic field is shown in blue, and the measurement terminals for the current and voltages I_{xx}, V_{xx} and V_{xy} are indicated. (b) Hall measurements with linear fits, allowing the carrier concentration to be determined. All measurements were made at ≈10 K.

Table S1: Electrical transport characteristics extracted from the data shown in Figure S2. The carrier concentration (n) and mobility (μ) are determined from the linear fits shown in Figure S2(b).

**δ-layer species, depth**	**Carrier concentration (n) ($10^{14}/\mathrm{cm}^2$)**	**Mobility (μ) (cm^2 / V s)**
As, z = 2 nm	N/A	N/A
As, 3 nm	0.22 ± 0.01	77 ± 2
P, 2 nm	0.82 ± 0.02	62.53 ± 0.04
P, 3 nm	1.42 ± 0.01	31.60 ± 0.01

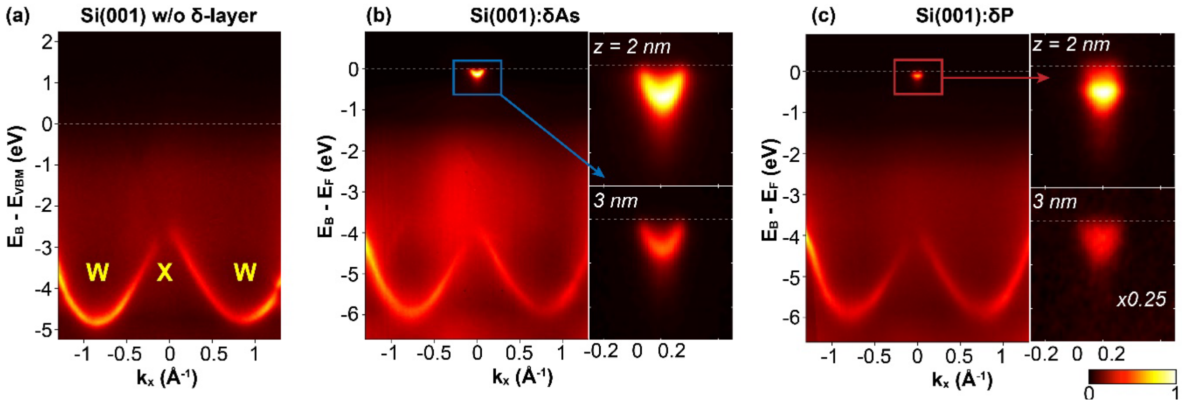

Figure S3: ARPES measurements along the XW symmetry line for samples with and without a δ-layer. (a) ARPES data for a Si(001) sample without a δ-layer, showing that there are no occupied conduction band states. The binding energy origin has been set to the valence band maximum. (b)-(c) ARPES data for a 2 nm deep (b) arsenic and (c) phosphorous δ-layer buried in Si(001). The binding energy origin is set to the Fermi-edge, where a zoom-in of the occupied conduction band is shown at two different depths. The colour scale is consistent across the figure and the data is take using a photon energy $h\nu$ = 380 eV.

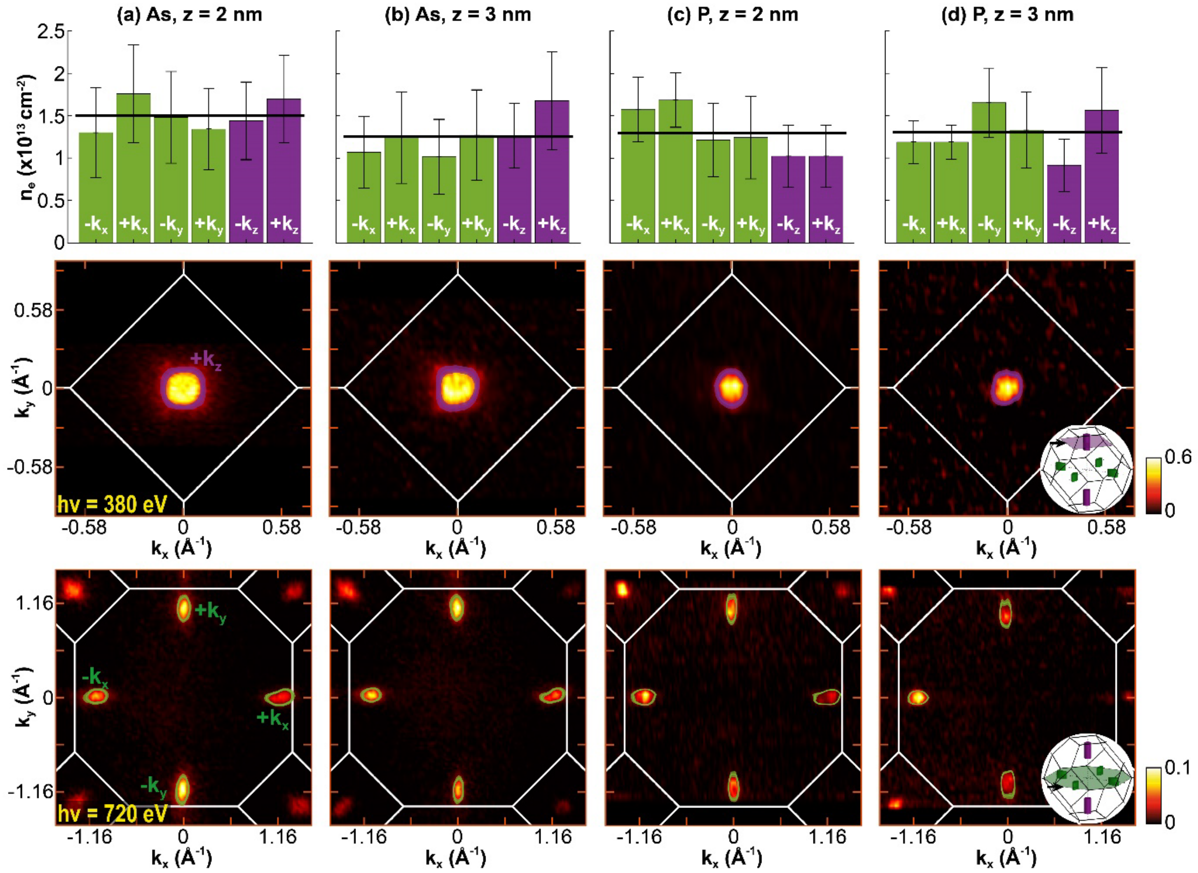

Figure S4: Summary of the SX-ARPES determined carrier concentration for each δ-layer sample. The dopant species and depth for each sample is labelled and ordered such that each column represents a summary of the data obtained; (a-b) arsenic and (c-d) phosphorous δ-layers buried (a,c) 2 nm and (b,d) 3 nm deep. (top) Breakdown of the carrier concentration for each of the six valleys, where the solid black line shows the average occupancy of each valley. (middle) ARPES iso-energetic slice extracted through k_x-k_y for the $+k_z$valley at $h\nu$ = 380 eV (bottom) ARPES iso-energetic slice extracted through k_x-k_y for the in-plane ($\pm k_x$,$\pm k_y$) valleys. The iso-slices are integrated over the binding energy, E_B, from -250 meV to E_F. The iso-contours are also shown, which show the best estimate for the total enclosed area for each valley.

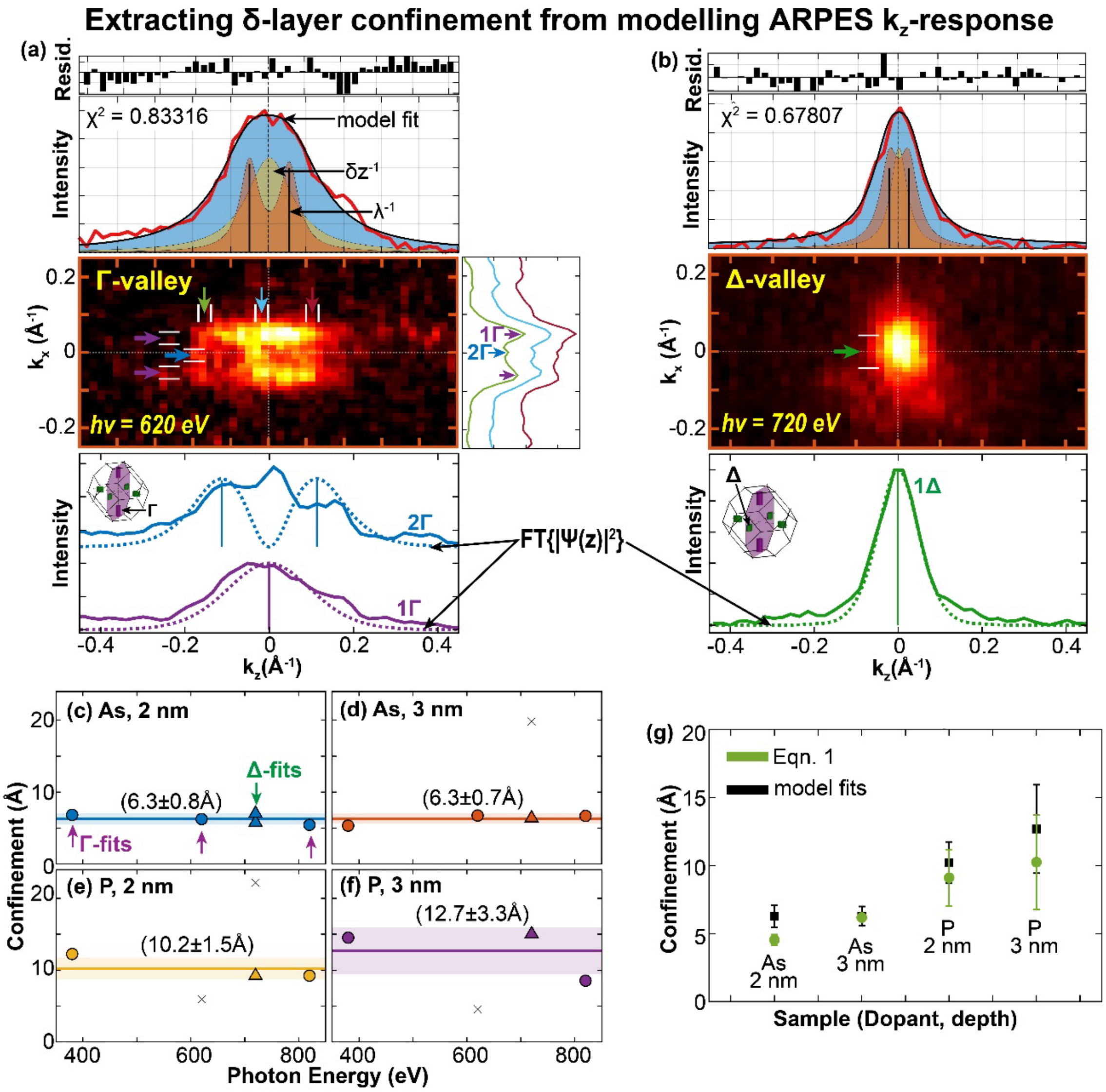

Figure S5: Extracting the δ-layer confinement from the ARPES k_z-response. (a) Example of the model fit for one Γ-valley dataset, whose chi-squared (χ^2) value is stated: (middle) k_x-k_z Fermi-surface; (upper) the k_x-integrated line-profile which shows the best fit (blue) to the data (red) by convolving the MFP-broadened (orange) and confinement-broadened (yellow) Lorentzian's; (lower) k_z and (right) k_x line profile cuts taken at three different points and colour-coded by the arrows. The white lines indicate the k-integration windows for the horizontal and vertical line-cuts. In the lower k_z line profiles, the Fourier Transform (FT) of the probability density function solutions from a Schrödinger-Poisson model of δ-layers (Figure S7) is in good agreement to the data. (b) The same as (a), but for a Δ-valley dataset. The ARPES k_z-response appears sharper since the 1Δ wave function is broader in real-space (see Figure S7). (c-f) Plot of the δ-layer confinement vs photon energy for the model fits defined in (a) and (b) for all samples. The circle and triangle data-points are from the Γ- and Δ-fits respectively. The black crosses indicate outlier fits; the k_z line profiles were noisy or had very poor fits to the data here. The shaded areas represent the uncertainty from the fitted range of values. (g) Comparison of the δ-layer confinement found using Equation 1 in the main manuscript (green) and the model fits (black) shown in (c-f). Both methods provide estimates to the δ-layer confinement that are in mutual agreement.

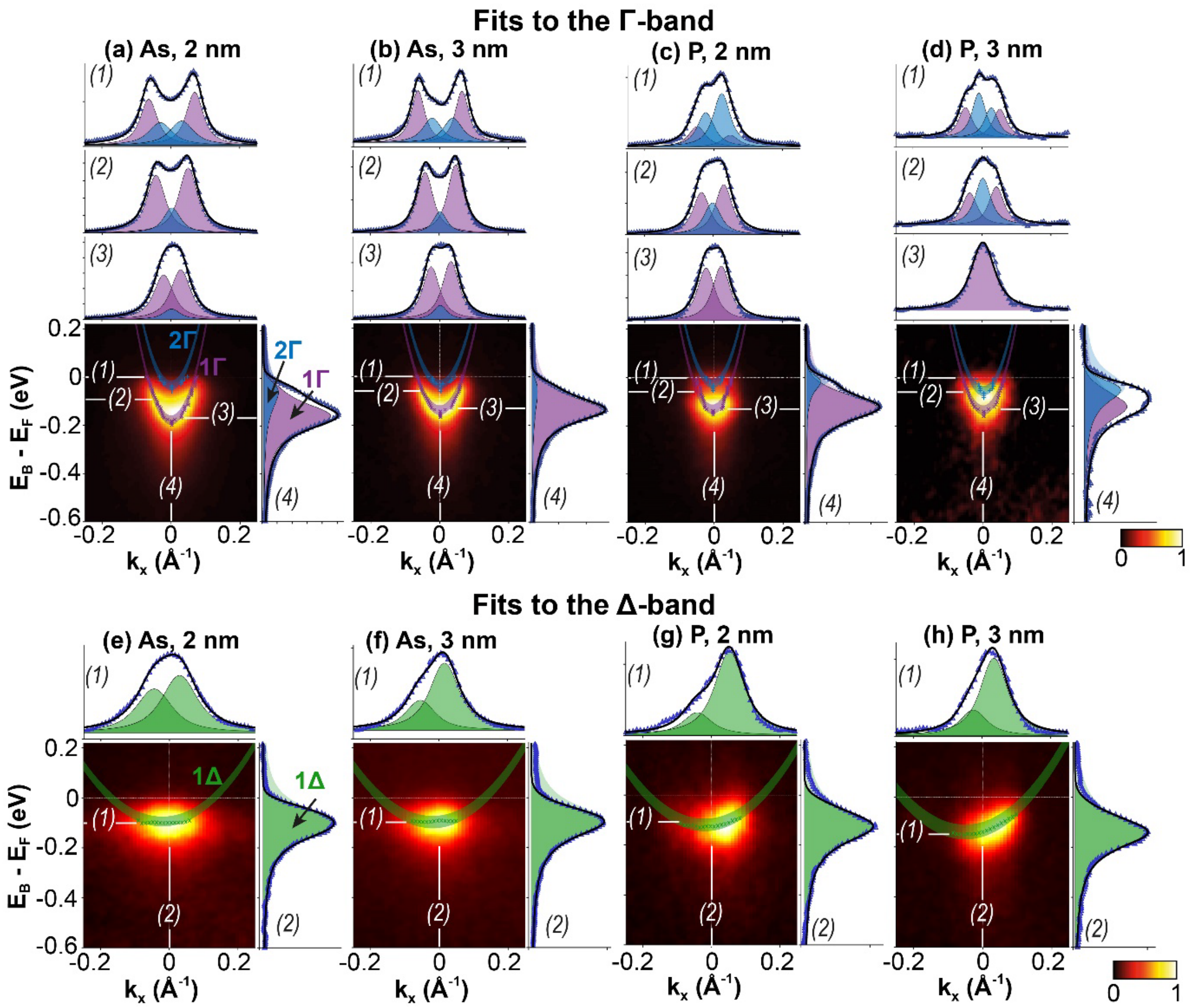

Figure S6: Summary of the ARPES curve fits to the (upper) Γ- and (lower) Δ-bands of the 2 and 3 nm arsenic and phosphorous δ-layer samples. The dopant species and depth for each sample is labelled and ordered such that each column represents a summary of the data and fits for each δ-layer sample. (a-d) Summary of the fits to the Γ-bands along the bulk XW symmetry line at a photon energy $h\nu$ = 380 eV. The purple and blue parabolas are the fits to the ARPES data, showing that the 1Γ and 2Γ states can be deconvolved. The line profiles above the ARPES image show the momentum distribution curves (MDCs) taken at three different binding energies, labelled (1)-(3); the line profile to the right, labelled (4) shows the corresponding fit to the energy distribution curve (EDC), taken through the centre of mass of the Γ-band. (e-h) Summary of the fits to the Δ-bands along the bulk ΓX symmetry line at a photon energy $h\nu$ = 720 eV. The green parabola shows the best fit to the 1Δ-band. The line profile above the ARPES image shows the fitted MDC, labelled (1) and the line profile to the right, labelled (2), shows a similarly fitted EDC, both of which are taken through the centre of mass of the Δ-band.

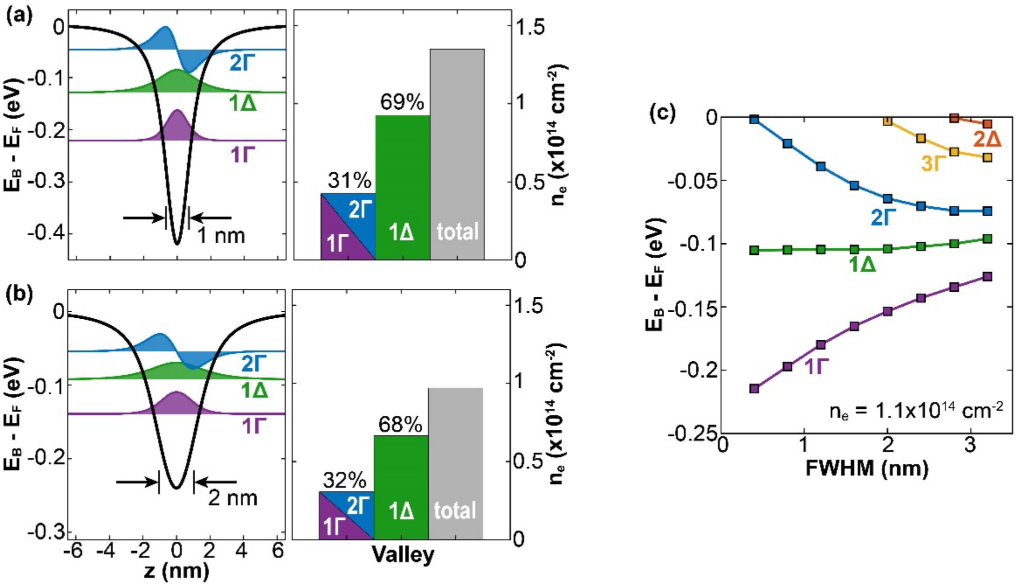

Figure S7: Summary of the solutions using a self-consistent Schrödinger-Poisson model of δ-layers. (a-b) Plot of the (left) wave-function and (right) carrier concentration solutions when using a charge distribution that has a FWHM of (a) 1 nm and (b) 2 nm. The occupancy of the Γ- and Δ-bands are shown as percentages, which show an equal occupancy (≈ 16%) for each sub-band. (c) Plot of the energy minima of each sub-band as a function of the FWHM of the charge distribution, using a δ-layer with a layer density of $1.1 \times 10^{14} \mathrm{cm}^{-2}$.
